# 

**Published:** 1857-10

**Authors:** 


### THE SANITARY REVIEW.

### CONTENTS OF No. XI.

The British Juggernaut in India . . . . . . 217
Vaccination . . . . . . . 239
Medical Geography and Endemic Diseases . . . . 249

**EPITOME OF SANITARY LITERATURE:**

Oesterlen's Handbook of Hygiene . . . . 260
Questions of the Day . . . . . . 261
Work, Fresh Air, and Exercise . . . . . *ib.*
Treatment of Fever by Fresh Air . . . . 262
Cholera in Lower Animals . . . . . 263
Life Assurance . . . . . . . *ib.*
Excessive Atmospheric Pressure the Cause of Cholera . . 264
Miscellaneous Works . . . . . . *ib.*

**ORIGINAL COMMUNICATIONS:**

Hygiene of the Turkish Army. By J. N. Radcliffe, Esq. (Concluded from p. 157) . . . . . . 265
Health of London during 1856. By John Webster, M.D., F.R.S. . 277
Summer Mourning for Females. By F. J. Brown, M.D. . . 286

**SANITARY AND SOCIAL SCIENCE.**

*Reports on Cities, Towns, and Districts.*—Teignmouth . . 288
M'Kinnell's Ventilator . . . . . . 294
City Dwellings for the Working Classes . . . . 296
Proposal for a London Female Sanitary Society and Savings' Bank . *ib.*

**PROGRESS OF EPIDEMICS in Forty-one Towns and Districts, contributed specially, by the Staff of Local Observers** . . . 297

**SANITARY LEGISLATION.**

The Board of Health: Medical Reform . . . . 315
National Association for the Promotion of Social Science . . *ib.*
Crimean Sanitary Commissioners' Report . . . . *ib.*
Report on Sheep and Contagious Diseases Prevention Bill . . 316
Netley Hospital . . . . . . . *ib.*

**TRANSACTIONS of the EPIDEMIOLOGICAL SOCIETY of LONDON:**

The Cholera Epidemy at the Island of Fogo, in the Cape de Verds, in 1855. By J. F. da Silva Leao . . . . 41
Historical Account of Gaol Fever. By F. C. Webb, M.D., F.S.A. . 63
Quarterly Report of Proceedings . . . . . 72

**NOTES AND CORRESPONDENCE.**

